# Literature Review on Health Emigration in Rare Diseases—A Machine Learning Perspective

**DOI:** 10.3390/ijerph20032483

**Published:** 2023-01-30

**Authors:** Małgorzata Skweres-Kuchta, Iwona Czerska, Elżbieta Szaruga

**Affiliations:** 1 Department of Organization and Management, Institute of Management, University of Szczecin, Cukrowa 8 Street, 71-004 Szczecin, Poland; 2 Department of Marketing Research, Faculty of Management, Wroclaw University of Economics and Business, 118/120 Komandorska Str, 53-345 Wroclaw, Poland; 3 Department of Transport Management, Institute of Management, University of Szczecin, Cukrowa 8 Street, 71-004 Szczecin, Poland

**Keywords:** health emigration, rare disease, orphan drug, access gap, discrimination in access to therapy

## Abstract

The article deals with one of the effects of health inequalities and gaps in access to treatments for rare diseases, namely health-driven emigration. The purpose of the paper is to systematize knowledge about the phenomenon of health emigration observed among families affected by rare diseases, for which reimbursed treatment is available, but only in selected countries. The topic proved to be niche; the issue of “health emigration in rare diseases” is an area for exploration. Therefore, the further analysis used text mining and machine learning methods based on a database selected based on keywords related to this issue. The results made it possible to systematize the guesses made by researchers in management and economic fields, to identify the most common keywords and thematic clusters around the perspective of the patient, drug manufacturer and treatment reimbursement decision-maker, and the perspective integrating all the others. Since the topic of health emigration was not directly addressed in the selected sources, the authors attempted to define the related concepts and discussed the importance of this phenomenon in managing the support system in rare diseases. Thus, they indicated directions for further research in this area.

## 1. Introduction

### 1.1. Presentation of Research Problems

The problem addressed in the article concerns one of the effects of unequal opportunities for families affected by rare diseases to access treatment for a narrow range of conditions for which there is a therapeutic option in the form of an orphan drug, and reimbursed treatment is only possible in selected countries around the world. As a result, some families choose to relocate and emigrate to a country where the patient’s health needs are likely to be met. 

The starting point for the article was a pre-diagnosed research gap in the field of health emigration in rare diseases after a literature review according to the keywords (a) “health emigration” and (b) “emigration” and “rare disease*”. Thus, the authors attempted to systematize socioeconomic and management knowledge around the issue of rare diseases in the context of unequal access to treatment, to capture potential gaps for further research directions, and to define concepts related to the research problem presented. 

The phenomenon of “health emigration” (named by the authors as emigration for a cure) is observed among patients affected by rare diseases, i.e., diseases that individually affect a small population. The definition of a rare disease, adopted in European Union countries, specifies the incidence of the disease at less than one person per 2000 inhabitants [1]. With a prevalence of 1 person per 50,000 population, the disease is defined as ultra-rare. The definition of rare (or ultra-rare) disease is not standardized globally (e.g., USA, Japan, Canada). Nevertheless, in each case, the rarity of the condition and the challenges it presents, especially in terms of the unmet health needs of patients, are emphasized. This thread is because rare diseases generally have a severe course and chronic nature and, in many cases, lead to the patient’s premature death. They are, therefore, particularly physically and psychologically burdensome not only for him, but also for his immediate family [2].

The scale of the problem is significant, as at least 6000 types of rare diseases have been defined so far, and it is estimated that they affect as much as 6–8% of the world’s population [3]. That is about 300 million people, among whom only 6% of medicine can offer a therapeutic option [4]. At the same time, it should be emphasized that a drug invented and approved for marketing does not always have a chance to reach the patient due to the countries’ different policies regarding reimbursement of drugs dedicated to rare diseases. Moreover, since these drugs—being innovative and produced on a small scale, usually with orphan drug status—most often tend to be horrendously expensive, their financing is problematic for limited public budgets and unattainable for the patient alone. As a result, because rare diseases mainly affect children, families choose to emigrate, with all its consequences [5]. 

For the sake of completeness, an orphan drug is the only or significantly more good product than an existing one for the diagnosis, prevention or treatment of a rare disease or other condition, for which marketing a medicinal product will not generate a sufficient return on investment. In Europe, this status, in principle, provides the drug manufacturer with ten years of market exclusivity in marketing medicinal products for a given indication [1,4]. 

Defining the phenomenon of health emigration in the literature is not apparent; hence the authors search and attempt to highlight this problem.

According to the Merriam-Webster online dictionary, emigration means leaving one’s residence, home or country to live or stay in another [6]. Investopedia, on the other hand, narrows the meaning of “emigration” to the relocation or process of people leaving one country to live in another [7]. The Cambridge English Dictionary gives a similar definition to the previous one but emphasizes the process of leaving a country “permanently” or living “permanently” in another country [8]. In turn, the process of people moving (usually en masse) to a new place of residence is called migration, according to the same dictionary [9]. It also represents the daily population movements associated with commuting to work or school [10]. Migration also has other meanings, not always related to humans. According to the Cambridge English Dictionary, it can mean, first, the movement of animals to another place—usually with a change of season. Second, migration can start using a new computer system and moving information, software or hardware from one computer system to another. Third, migration is also called the process of existing customers changing services or companies [9]. Two types of customer migration can be voluntary churn, i.e., the customer abandons the product, and involuntary churn, i.e., the supplier, discontinues the relationship with the customer. Forced migration often occurs due to a customer’s failure to pay fees or breach of contract [11].

Migration is either the result of environmental changes or the cause of their occurrence. According to this division, migration is either a dependent variable, determined by specific characteristics, or an independent variable focusing on its occurrence’s economic, social, political and natural consequences [12]. According to economic theories, one can primarily determine migration by economic variables. Sociological theories, on the other hand, focus on the motives behind migration decisions and are based on behaviourist research. Finally, geographic theories analyze the pull factors and barriers to migration in spatially diverse environments [12]. According to Wojciech Janicki, none of the migration theories developed so far can comprehensively and convincingly explain the variability of complex migration processes. This author argues that the universalization of migration theory would require coordinating interdisciplinary research conducted by specialists from different scientific disciplines [12].

Another concept related to movement is immigration. According to the PWN Dictionary of the Polish Language, immigration means an influx of foreign people into a country to settle there, and a foreign population settled in some country [13]. A more expansive definition of immigration is leaving one’s own country and moving to another country of which one is not an indigenous resident or citizen in order to [14]:

- Settle or live there (as a permanent resident or naturalized citizen);
- Take up employment as a migrant worker;
- Live temporarily as a foreign worker.

To summarise, emigration and immigration are types of external migration, i.e., foreign migration associated with crossing national borders. Within the framework of external migration, other types of migration can also be distinguished [15]:

- Re-emigration, or the return of emigrants to the country after a permanent stay abroad;
- Repatriation, i.e., the mass return to the country, organized by state authorities, of prisoners of war, internees, people who left the country, e.g., for political reasons;
- Deportation, i.e., forced expulsion from the national territory, most often having to do with illegal immigrants;
- Expatriation is the voluntary or forced departure from one’s national territory, signifying a break from the country.

In addition to external migration, it is necessary to mention the second—internal migration, i.e., taking place within a country. Here, there is a change of residence within a country—inter-regional and intra-regional migration can occur [15].

A concept also related to the process of movement is refugeeism. Refugees are people who flee their country to another country to avoid some conflict/problem. The term refugee also includes asylum seekers, i.e., people forced to leave their country to avoid persecution—often because of political or religious beliefs. Internally displaced persons forced to leave their homes and move to a new place in their country are also considered refugees [16].

The strongest determinants of migration processes at present, in the context of the sizeable interregional variation in the economic situation, are motives of an economic nature and the propensity to improve living conditions [15]. Other determinants of migration processes are family, education, religion, politics, nationality, health, leisure, and climate [15,17,18,19]. According to Nicole B. Simpson, the key predictors of migration are income disparity, migrant networks and demographic factors (age, education, marital status, and language) [20]. Studies show that public spending by home country governments on free public education, access to health care and unemployment benefits reduces international emigration [21].

The phenomenon of “health emigration” refers to a situation in which a family seeks the most advanced treatment abroad for their sick child [22]. Health emigration is believed to be getting rid of citizens, both parents who have a sick child and that child, but also healthy siblings. In doing so, the author draws attention to the disturbing profile of these emigrant parents in the context of abandoning their country of origin: they are educated, working-age persons who will eventually work for the economy and development of another country [23]. Health emigration is an effect of desperation. If one receives information about the lack of funding for treatment in a particular country and the diagnosis of an illness, attachment to the home country goes down the drain [24]. 

Machine learning is being applied to studying health emigration in the context of rare diseases. It is used, for example, for literature review. In addition, machine learning will shortly support medical professionals in diagnosing diseases [25].

Machine learning (ML) is the scientific study of algorithms and statistical models used by computer systems to perform a specific task without explicit programming [26]. It represents a growing branch of computational algorithms in emulating human intelligence by learning from the surrounding environment [27]. Machine learning helps decision-making through prediction and classification mechanisms based on historical data [28]. In general, ML algorithms are designed to perform two main tasks: supervised and unsupervised learning. In supervised learning, the sample contains both input and output indicators. In this case, ML algorithms build a rule, and the input indicator is mapped to the output using the rule. In unsupervised learning, on the other hand, the samples have no original labels, which should be understood as the absence of output indicators [29]. 

In conclusion, machine learning has potential and offers excellent opportunities in the social sciences. In addition, technological advances are making machine learning tools an attractive alternative to classical methods, and the technical barriers to using ML are decreasing thanks to available open-source software [30]. 

### 1.2. Organization of the Paper

The article aims to systematize knowledge on health emigration among families affected by rare diseases through a literature review using machine learning and an analysis of available industry reports. 

In order to achieve the goal, the following research questions were formulated:

1. Define the basic concepts related to the topics of the article: emigration and rare diseases.
2. What socioeconomic and management issues dominate scientific analysis in the area of unequal access to the treatment of rare diseases?
3. To what extent does machine learning help identify the state of knowledge and research on health emigration in rare diseases and its limitations?
4. For what reasons is the phenomenon of health emigration relevant to the management strategy of the support system in rare diseases?

The structure of the article corresponds to the objectives set. The introduction describes the genesis, research problem, and basic definitions related to the phenomenon of emigration, including that of health emigration observed in rare diseases. A literature review of socioeconomic issues related to rare diseases, orphan drugs, treatment and access to treatment using a machine learning tool was conducted. Unequal access to treatment with orphan drugs is a direct cause of health emigration in rare diseases, hence this choice of keywords. The discussion develops emigration’s theme from the point of view of its relevance to rare disease management strategies. The article closes with conclusions and an indication of the limitations of the analyzes conducted and potential research directions. 

## 2. Materials and Methods

Implementing the article’s aim required a specific research procedure regarding sources and methodology. The authors have based the study on a literature review on access to treatment for rare diseases. The bibliography includes 343 works, among them scientific articles, books, specialist literature, and electronic sources from 1989–2022, and among these works, the most were those from 2017–2021. During desk research analysis, the authors have used the following professional scientific databases: Scopus, the Web of Science, and PubMed. The authors used these scientific databases due to their possible access by having an account. Secondly, these databases made it possible to complete the literature for this article. The authors wanted to show scientists’ contributions to social and health sciences development through these databases. 

All publications were searched on the Web of Science [31], Scopus [32], and PubMed [33] databases. The keywords sequence used for article selection was: orphan* and “rare disease*” and treatment* and access*.

In the next phase of literature selection, the duplicate was removed, and the content was checked to match the topic. Many valuable publications in other languages or indexed in other databases, or those not indexed, were not included in the study. Due to the necessity of tokenization and transformation of texts (including lemmatization), similar restrictions had to be adopted. Ultimately, 343 publications in English were selected for the analysis of text mining and machine learning.

The analysis used techniques in the field of machine learning and mining text such as word cloud [34,35], mapping latent variables with network connections [36,37], a bag of words [38,39], topic modeling by Latent Semantic Index (LSI) [39,40], emotion recognition (profiler by Plutchik emotions) [41,42] and geomapping [43].

**Figure 1:**
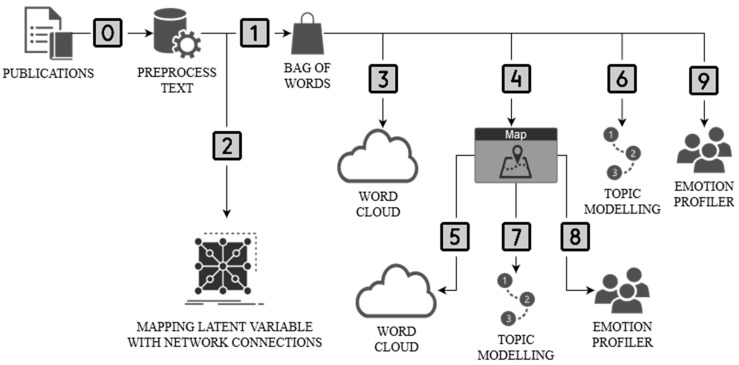
The framework of the authors’ research procedure (methodology). Source: own elaboration.

The research procedure consisted of 10 stages (Figure 1). The preprocessed text was performed in stage zero. This stage focused on the transformation (lowercase, remove URLs) and tokenization of the text (regexp) as well as its normalization (Lemmagen Lematizer) and filtering (stopwords, lexicon, regexp). The Bag of Words (term frequency—count, regularization—L2 (Euclidean)) was used in the first stage. Step 2 identifies latent variables in the term relationship network map. Stages 3–6 were carried out in parallel. In step 3, a word cloud was created for all publications. Then, in stage 4, these publications were geomapped based on abstracts. In step 5, a word cloud was created for geographic locations. In step 6, the Latent Semantic Index was used for topic modeling. Based on the collected information, the topic modeling (LSI) results were geomapped with a selection of two cases: Poland and Brazil (stage 7). These results were enriched with emotion profiling (recognition of emotions) in publications using (Plutchik’s profile) for the case of Poland and Brazil (stage 8). The last stage (9th) is an extension of stage 8 by analyzing the range of emotions for two keywords: access and immigration.

## 3. Results

**Figure 2:**
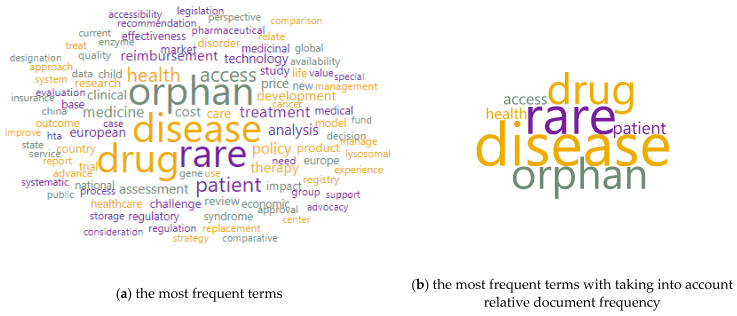
Word clouds of terms. Limitations: the authors did not take into account many valuable publications in the study, instead taking into account the need to narrow the thematic scope of the described issues. Source: own elaboration based on 343 references [44,45,46,47,48,49,50,51,52,53,54,55,56,57,58,59,60,61,62,63,64,65,66,67,68,69,70,71,72,73,74,75,76,77,78,79,80,81,82,83,84,85,86,87,88,89,90,91,92,93,94,95,96,97,98,99,100,101,102,103,104,105,106,107,108,109,110,111,112,113,114,115,116,117,118,119,120,121,122,123,124,125,126,127,128,129,130,131,132,133,134,135,136,137,138,139,140,141,142,143,144,145,146,147,148,149,150,151,152,153,154,155,156,157,158,159,160,161,162,163,164,165,166,167,168,169,170,171,172,173,174,175,176,177,178,179,180,181,182,183,184,185,186,187,188,189,190,191,192,193,194,195,196,197,198,199,200,201,202,203,204,205,206,207,208,209,210,211,212,213,214,215,216,217,218,219,220,221,222,223,224,225,226,227,228,229,230,231,232,233,234,235,236,237,238,239,240,241,242,243,244,245,246,247,248,249,250,251,252,253,254,255,256,257,258,259,260,261,262,263,264,265,266,267,268,269,270,271,272,273,274,275,276,277,278,279,280,281,282,283,284,285,286,287,288,289,290,291,292,293,294,295,296,297,298,299,300,301,302,303,304,305,306,307,308,309,310,311,312,313,314,315,316,317,318,319,320,321,322,323,324,325,326,327,328,329,330,331,332,333,334,335,336,337,338,339,340,341,342,343,344,345,346,347,348,349,350,351,352,353,354,355,356,357,358,359,360,361,362,363,364,365,366,367,368,369,370,371,372,373,374,375,376,377,378,379,380,381,382,383,384,385,386].

Figure 2 shows word clouds based on the examined corpus, which included the title and keywords. It illustrates the most common terms around the studied issue. In the initial examination, it could be noticed that the most important words were: disease (weight 406), rare (weight 351), an orphan (weight 283), drug (weight 281), patient (weight 112), access (weight 102), health (weight 98). In a tag cloud, the weight is the same as the number of occurrences.

**Figure 3:**
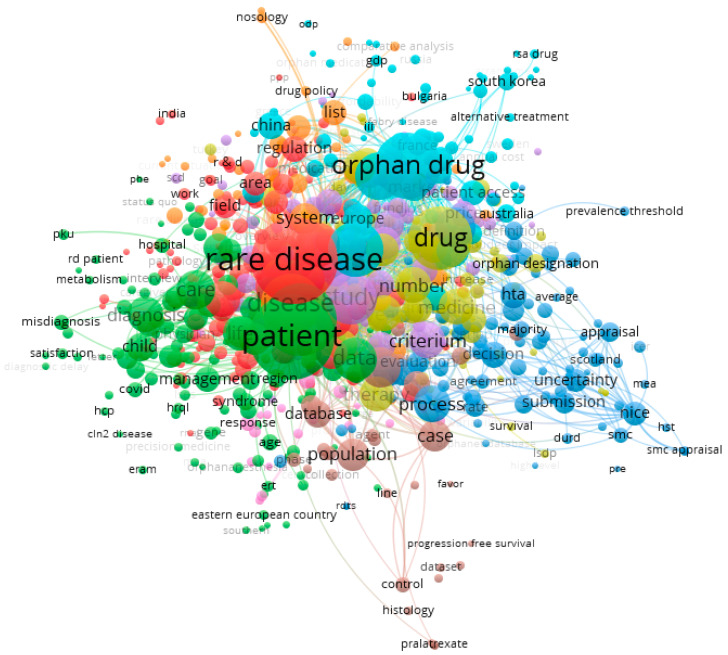
Mapping latent variables from titles and abstracts of the publications on issues connected with rare diseases. Note: Technical publication on mapping and clustering using VOSviewer [36,37]. Limitations: the authors did not take into account many valuable publications in the study, instead taking into account the need to narrow the thematic scope of the described issues. Source: own elaboration based on 343 references [44,45,46,47,48,49,50,51,52,53,54,55,56,57,58,59,60,61,62,63,64,65,66,67,68,69,70,71,72,73,74,75,76,77,78,79,80,81,82,83,84,85,86,87,88,89,90,91,92,93,94,95,96,97,98,99,100,101,102,103,104,105,106,107,108,109,110,111,112,113,114,115,116,117,118,119,120,121,122,123,124,125,126,127,128,129,130,131,132,133,134,135,136,137,138,139,140,141,142,143,144,145,146,147,148,149,150,151,152,153,154,155,156,157,158,159,160,161,162,163,164,165,166,167,168,169,170,171,172,173,174,175,176,177,178,179,180,181,182,183,184,185,186,187,188,189,190,191,192,193,194,195,196,197,198,199,200,201,202,203,204,205,206,207,208,209,210,211,212,213,214,215,216,217,218,219,220,221,222,223,224,225,226,227,228,229,230,231,232,233,234,235,236,237,238,239,240,241,242,243,244,245,246,247,248,249,250,251,252,253,254,255,256,257,258,259,260,261,262,263,264,265,266,267,268,269,270,271,272,273,274,275,276,277,278,279,280,281,282,283,284,285,286,287,288,289,290,291,292,293,294,295,296,297,298,299,300,301,302,303,304,305,306,307,308,309,310,311,312,313,314,315,316,317,318,319,320,321,322,323,324,325,326,327,328,329,330,331,332,333,334,335,336,337,338,339,340,341,342,343,344,345,346,347,348,349,350,351,352,353,354,355,356,357,358,359,360,361,362,363,364,365,366,367,368,369,370,371,372,373,374,375,376,377,378,379,380,381,382,383,384,385,386].

**Figure 4:**
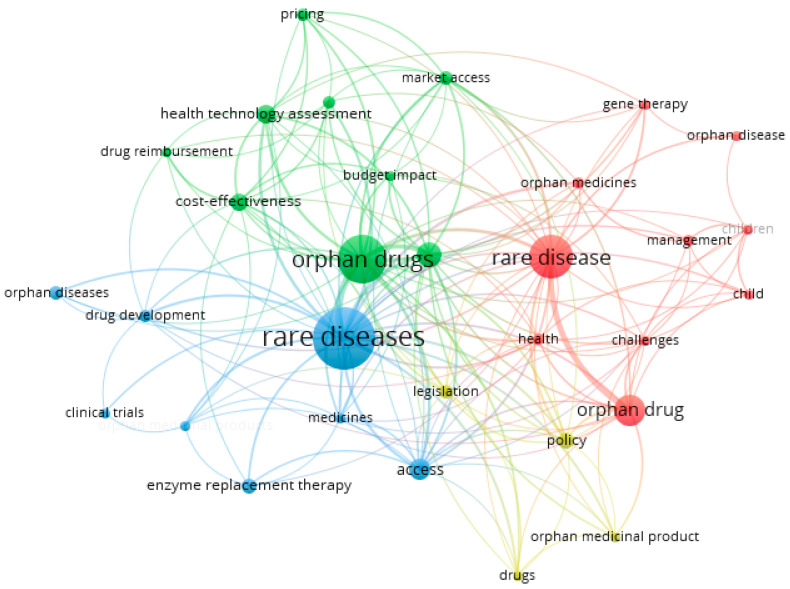
Mapping latent variables from keywords of the publications on issues connected with rare diseases. Note: Technical publication on mapping and clustering using VOSviewer [36,37]. Limitations: the authors did not take into account many valuable publications in the study, instead taking into account the need to narrow the thematic scope of the described issues. Source: own elaboration based on 343 references [44,45,46,47,48,49,50,51,52,53,54,55,56,57,58,59,60,61,62,63,64,65,66,67,68,69,70,71,72,73,74,75,76,77,78,79,80,81,82,83,84,85,86,87,88,89,90,91,92,93,94,95,96,97,98,99,100,101,102,103,104,105,106,107,108,109,110,111,112,113,114,115,116,117,118,119,120,121,122,123,124,125,126,127,128,129,130,131,132,133,134,135,136,137,138,139,140,141,142,143,144,145,146,147,148,149,150,151,152,153,154,155,156,157,158,159,160,161,162,163,164,165,166,167,168,169,170,171,172,173,174,175,176,177,178,179,180,181,182,183,184,185,186,187,188,189,190,191,192,193,194,195,196,197,198,199,200,201,202,203,204,205,206,207,208,209,210,211,212,213,214,215,216,217,218,219,220,221,222,223,224,225,226,227,228,229,230,231,232,233,234,235,236,237,238,239,240,241,242,243,244,245,246,247,248,249,250,251,252,253,254,255,256,257,258,259,260,261,262,263,264,265,266,267,268,269,270,271,272,273,274,275,276,277,278,279,280,281,282,283,284,285,286,287,288,289,290,291,292,293,294,295,296,297,298,299,300,301,302,303,304,305,306,307,308,309,310,311,312,313,314,315,316,317,318,319,320,321,322,323,324,325,326,327,328,329,330,331,332,333,334,335,336,337,338,339,340,341,342,343,344,345,346,347,348,349,350,351,352,353,354,355,356,357,358,359,360,361,362,363,364,365,366,367,368,369,370,371,372,373,374,375,376,377,378,379,380,381,382,383,384,385,386].

While exploring the studied publications in-depth, particular attention was paid to mapping latent variables in the network connections map (Figure 3 and Figure 4). The larger the ball, the more often the latent variable in the analyzed texts appeared. The shorter the bond line, the stronger the bond, and the longer, the weaker the interdependence of the latent variables.

Based on the titles and abstracts of the analyzed publications, 9189 terms (latent variables) were found, 746 of which appeared five times in the text. On their basis, using the “association length” method, nine clusters were found in the connection network, with a total number of 96,778 connections (Figure 3):

- Cluster 1—red (149 items);
- Cluster 2—green (138 items);
- Cluster 3—blue (104 items);
- Cluster 4—yellow (92 items);
- Cluster 5—purple (82 items);
- Cluster 6—celeste (74 items);
- Cluster 7—orange (47 items);
- Cluster 8—brown (34 items);
- Cluster 9—pink (26 items).

Using bibliographic data and analyzing only “keywords”, 31 keywords were selected (out of 1724) that appeared at least five times in the text (full counting). Four clusters with 174 connections were obtained (Figure 4):

- Cluster 1 (red) includes 10 latent variables: challenges, child, children, gene therapy, health, management, orphan disease, orphan drug, orphan medicines, and rare disease.
- In cluster 2 (green), there are nine latent variables: budget impact, cost-effectiveness, drug reimbursement, health policy, health technology assessment, market access, orphan drug, pricing, and reimbursement.
- Cluster 3 (blue) includes seven latent variables: access, clinical trials, drug development, enzyme replacement therapy, medicines, orphan diseases, and rare disease.
- Cluster 4 (yellow) includes four latent variables: drugs, legislation, orphan medicine product, and policy.

Individual clusters can be assigned a perspective that seems to bind together the keywords extracted within it. Thus, cluster 1 is the perspective of the patient; cluster 2 is the perspective of the policy maker/payer responsible for reimbursement in a given country; cluster 3 is the perspective of the researcher/inventor/manufacturer of the drug, while cluster 4 may bring together all stakeholders in international agreements and solutions to better meet the needs of rare disease patients. All perspectives naturally intersect, which only underscores the need for inclusive and acceptable solutions for all parties.

**Figure 5:**
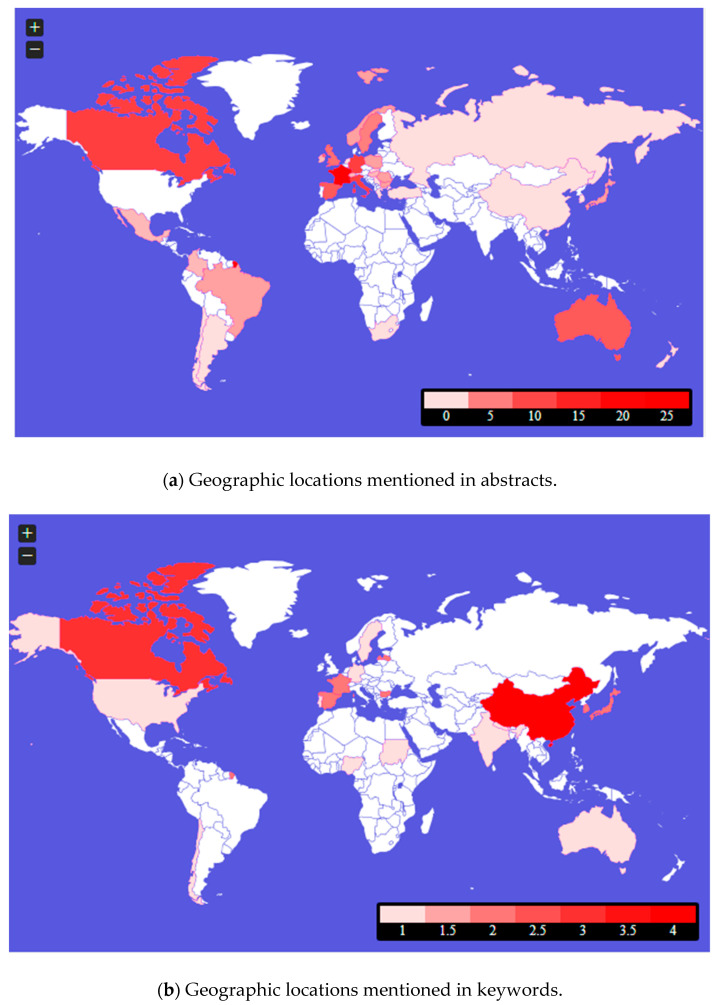
Publications’ content maps from abstracts and keywords. Note: the more intense the color in the legend, the more often the geographic location is mentioned. Source: own elaboration based on 343 references [44,45,46,47,48,49,50,51,52,53,54,55,56,57,58,59,60,61,62,63,64,65,66,67,68,69,70,71,72,73,74,75,76,77,78,79,80,81,82,83,84,85,86,87,88,89,90,91,92,93,94,95,96,97,98,99,100,101,102,103,104,105,106,107,108,109,110,111,112,113,114,115,116,117,118,119,120,121,122,123,124,125,126,127,128,129,130,131,132,133,134,135,136,137,138,139,140,141,142,143,144,145,146,147,148,149,150,151,152,153,154,155,156,157,158,159,160,161,162,163,164,165,166,167,168,169,170,171,172,173,174,175,176,177,178,179,180,181,182,183,184,185,186,187,188,189,190,191,192,193,194,195,196,197,198,199,200,201,202,203,204,205,206,207,208,209,210,211,212,213,214,215,216,217,218,219,220,221,222,223,224,225,226,227,228,229,230,231,232,233,234,235,236,237,238,239,240,241,242,243,244,245,246,247,248,249,250,251,252,253,254,255,256,257,258,259,260,261,262,263,264,265,266,267,268,269,270,271,272,273,274,275,276,277,278,279,280,281,282,283,284,285,286,287,288,289,290,291,292,293,294,295,296,297,298,299,300,301,302,303,304,305,306,307,308,309,310,311,312,313,314,315,316,317,318,319,320,321,322,323,324,325,326,327,328,329,330,331,332,333,334,335,336,337,338,339,340,341,342,343,344,345,346,347,348,349,350,351,352,353,354,355,356,357,358,359,360,361,362,363,364,365,366,367,368,369,370,371,372,373,374,375,376,377,378,379,380,381,382,383,384,385,386].

Figure 5 is the publications’ content maps. They include geographical locations (probably country cases, country clinical trials, or country system solutions) mentioned in the abstracts and keywords of the analyzed publications. These threads require further in-depth analysis, set in the context of the genetic determinants of the area on the one hand and the health policies of individual countries on the other.

Figure 5 shows which locations were most often referred to by the authors in the context of rare diseases and medical availability. The abstracts show that such countries were: France, Germany, Canada, Italy, Australia, and Great Britain. There were also countries such as Mexico, Columbia, Brazil, Argentina, Chile, South Africa, Spain, Ireland, Switzerland, the Netherlands, Poland, Sweden, Norway, Slovakia, Hungary, Serbia, Romania, Bulgaria, Greece, Turkey, Russia, China, Korea, Japan, and New Zealand. However, in keywords, they most often referred to countries such as China and Canada, Japan, Korea, Bulgaria, Latvia, France, and Spain. There were also countries such as the United States, Chile, Nigeria, Sudan, Portugal, Germany, Sweden, India, Nepal, and Australia.

**Figure 6:**
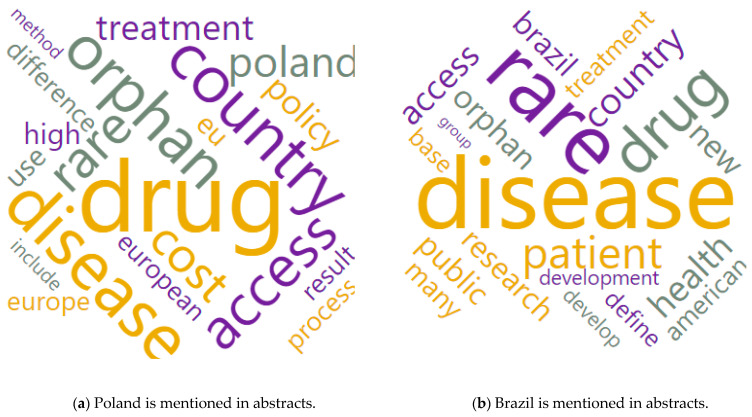
Word clouds of terms from abstracts connected with geographic locations. Limitations: the authors did not take into account many valuable publications in the study, instead taking into account the need to narrow the thematic scope of the described issues. Source: own elaboration based on 343 references [44,45,46,47,48,49,50,51,52,53,54,55,56,57,58,59,60,61,62,63,64,65,66,67,68,69,70,71,72,73,74,75,76,77,78,79,80,81,82,83,84,85,86,87,88,89,90,91,92,93,94,95,96,97,98,99,100,101,102,103,104,105,106,107,108,109,110,111,112,113,114,115,116,117,118,119,120,121,122,123,124,125,126,127,128,129,130,131,132,133,134,135,136,137,138,139,140,141,142,143,144,145,146,147,148,149,150,151,152,153,154,155,156,157,158,159,160,161,162,163,164,165,166,167,168,169,170,171,172,173,174,175,176,177,178,179,180,181,182,183,184,185,186,187,188,189,190,191,192,193,194,195,196,197,198,199,200,201,202,203,204,205,206,207,208,209,210,211,212,213,214,215,216,217,218,219,220,221,222,223,224,225,226,227,228,229,230,231,232,233,234,235,236,237,238,239,240,241,242,243,244,245,246,247,248,249,250,251,252,253,254,255,256,257,258,259,260,261,262,263,264,265,266,267,268,269,270,271,272,273,274,275,276,277,278,279,280,281,282,283,284,285,286,287,288,289,290,291,292,293,294,295,296,297,298,299,300,301,302,303,304,305,306,307,308,309,310,311,312,313,314,315,316,317,318,319,320,321,322,323,324,325,326,327,328,329,330,331,332,333,334,335,336,337,338,339,340,341,342,343,344,345,346,347,348,349,350,351,352,353,354,355,356,357,358,359,360,361,362,363,364,365,366,367,368,369,370,371,372,373,374,375,376,377,378,379,380,381,382,383,384,385,386].

Figure 6 presents word clouds again, but in the context of spatial differentiation. The case of Poland and Brazil, which had the same number of presentations in the abstracts, was taken at random.

Figure 6 shows that the case of Poland was characterized by an orientation to the European market, medical availability in terms of costs, differences in treatment methods, results, treatment process, and the sector policy itself. On the other hand, in the case of Brazil, the focus was mainly on the American market, development, the role of the patient, access to public goods, and research development. The economic difference between these cases may lead to the supposition that in the case of Brazil, the threads focus on the availability of public health care in the case of rare diseases and the case of Poland—on the cost (e.g., from private funds) of such care. Two different approaches may be the aftermath of the policy of financing the treatment of rare diseases (or even the lack thereof), the level of economic development of the country, and the wealth of the society (measured by GDP/capita purchasing power parity (PPP)) and the necessity of health emigration.

Based on Table 1, five topics were identified, which are shaped by keywords. Topic modeling allows the discovery of research issues based on clusters of words from each publication (document). One document can contain many subjects with different relationships and proportions. Among them, keywords have been distinguished that strongly represent the topic and those that correspond to it to a lesser extent (links are not transparent). For example, if the text contains many references to China, HTA, policy, rare, insurance, and country, and no reference to a product, designation, FDA, or approval, it falls under topic 4. If the text only refers to rare diseases, care, patient, and R&D sphere, but does not refer to costs, price, drugs, HTA, or orphan, it belongs to topic 2. Based on the topic consistency score = 0.3841, it can be concluded that it is good but moderate topic consistency in 343 publications. This conclusion is consistent with the conclusion from the latent variable mapping (Figure 3 and Figure 4). It was also the basis for narrowing down the topic modeling criteria.

Topic modeling, taking into account an additional criterion, i.e., referring to the country in the abstract, is presented in Table 2.

Table 2 shows 3 thematic groups for selected cases: Poland and Brazil. For example, if the text frequently refers to such terms as drug, country, disease, orphan, requirement, rare, access, GDP, MCDA, and HTA, it represents the first thematic group (the case of Poland). However, if in the text there are many references to GDP per capita on an annual basis to costs but without the words, MCDA, OD, Russia, criterion, or world, the publications represent the 2nd thematic group (the Polish case). In the case of frequent reference to the words: drug, regard, LA, market, Latin, orphan, and EU, but excluding the words: policy, patient, and company, the topic represents the 3rd thematic group for the Brazil case. The simultaneous appearance of the words: disease, rare, policy, country, patient, legislation, Latin, orphan, and clinical is characteristic of the modeling of the first group of the topic for the case of Brazil. Narrowing the reconnaissance by considering an additional (geographical) criterion increases topic consistency, which allows us to conclude that geographic location changes the optics of the approach to access to treatment in rare diseases. In publications where Brazil was mentioned, there is greater topic coherence in this area than in Poland (slight difference).

**Figure 7:**
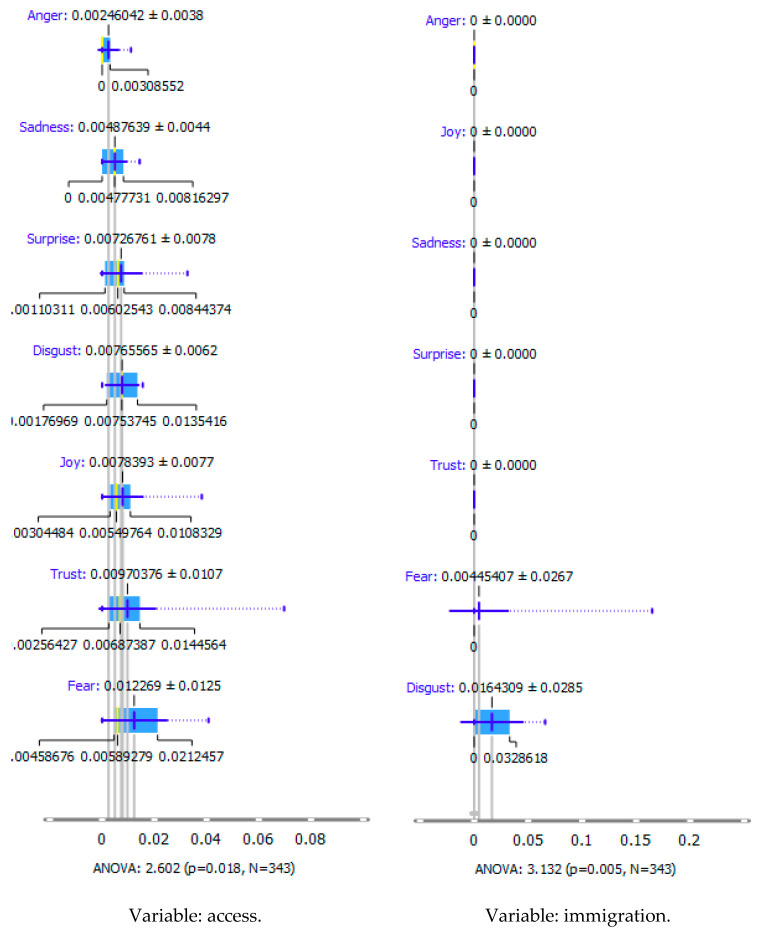
Emotion recognition for health migration in rare diseases with access and immigration. Source: own elaboration based on 343 references [44,45,46,47,48,49,50,51,52,53,54,55,56,57,58,59,60,61,62,63,64,65,66,67,68,69,70,71,72,73,74,75,76,77,78,79,80,81,82,83,84,85,86,87,88,89,90,91,92,93,94,95,96,97,98,99,100,101,102,103,104,105,106,107,108,109,110,111,112,113,114,115,116,117,118,119,120,121,122,123,124,125,126,127,128,129,130,131,132,133,134,135,136,137,138,139,140,141,142,143,144,145,146,147,148,149,150,151,152,153,154,155,156,157,158,159,160,161,162,163,164,165,166,167,168,169,170,171,172,173,174,175,176,177,178,179,180,181,182,183,184,185,186,187,188,189,190,191,192,193,194,195,196,197,198,199,200,201,202,203,204,205,206,207,208,209,210,211,212,213,214,215,216,217,218,219,220,221,222,223,224,225,226,227,228,229,230,231,232,233,234,235,236,237,238,239,240,241,242,243,244,245,246,247,248,249,250,251,252,253,254,255,256,257,258,259,260,261,262,263,264,265,266,267,268,269,270,271,272,273,274,275,276,277,278,279,280,281,282,283,284,285,286,287,288,289,290,291,292,293,294,295,296,297,298,299,300,301,302,303,304,305,306,307,308,309,310,311,312,313,314,315,316,317,318,319,320,321,322,323,324,325,326,327,328,329,330,331,332,333,334,335,336,337,338,339,340,341,342,343,344,345,346,347,348,349,350,351,352,353,354,355,356,357,358,359,360,361,362,363,364,365,366,367,368,369,370,371,372,373,374,375,376,377,378,379,380,381,382,383,384,385,386].

An extension of the above analysis is the recognition (profiling) of emotions according to the Plutchik classification (Table 3 and Figure 7).

As shown in Table 3, the content of publications on migration due to rare diseases has a dynamic character and carries an emotional charge with different markings (positive and negative). For the most part, however, the positive dominate over the negative. When profiling emotions in publications where Poland was mentioned in the abstract, joy dominates over trust in a 4: 1 ratio, but these are positive emotions. However, where Brazil has been mentioned, there are more emotions: trust, surprise, sadness, and fear. Positive emotions versus negative emotions are 2:1. In the case of the analysis of all publications, where the abstract refers to any geographical location, the profile of emotions is more diverse, with the ratio of positive to negative emotions being approximately 4:1. By digging into the data, it can be identified the words that are the creators of these emotions in the message of content and define the scope.

During reading publications, it can be concluded that the term “access” in this issue evokes seven emotions, from positive to negative, which statistically differs from each other. “Access” carries a load of emotions, such as fear, trust, joy, disgust, surprise, sadness, and anger. Another term that evokes emotions and is statistically significant from the point of view of profiling emotions is “immigration”. This term evokes negative emotions: fear and disgust. The emotion of the word “emigration” could not be recognized. The issue of immigration has appeared in publications in the context of studying the regularities characteristic of migrant communities and their impact on the solutions offered/postulated in the host country. 

Summing up, it can be said that the most significant ranges of emotions were recorded for the word access, such as fear, trust, and disgust. The most significant deviation (and the coefficient of variation) is that of trust. In the case of immigration, the more fantastic range of the scale of emotions is “disgust”, and the greater the coefficient of variation is “fear”. The word immigration does not evoke positive emotions: trust, surprise, joy, but also does not carry negative feelings such as sadness and anger in these publications.

## 4. Discussion

The literature has turned out to be scarce in the threads devoted to health emigration in the area of rare diseases. The scientific and expert communities discuss health inequalities, gaps in access to treatment, problems with reimbursement for orphan drugs and the need for change. According to the articles’ authors, it is worth filling this research gap. After all, emigration for medicine, dubbed “health emigration” by the authors, raises several micro and macro consequences—for the family in question and the system [386,387]. Moreover, they cite these arcane arguments as part of the discussion. 

Although the problem still affects a narrow range of people, its importance is significant since a possible solution will stimulate the development of other much-needed orphan therapies. However, the process requires cross-state and cross-sectoral agreements [388,389,390,391]. The efforts of the last two decades, made internationally and by individual countries to eliminate restrictions and fight the exclusion of this group of societies, have brought many positive changes [389]. However, disparities in the implementation of pro-patient solutions and funding for rare disease treatment will exacerbate the phenomenon of health emigration [392]. Emigration, while paradoxically solving the individual patient’s problem, distorts the pattern of systemic solutions. If the patient (or his or her family) finds a solution in another country, the other stakeholders (the drug manufacturer and the reimbursement decision maker in the country of origin) have no incentive to seek a reimbursement compromise for the original outlet. 

The incentive system for the science and technology sector has significantly increased the number of new therapies. Between 2010 and 2021, six hundred and sixty new molecules previously approved by the EMA were launched on the EU market, 19% of which were orphan products [392]. Orphan products are no longer a niche; they are one of the fastest-growing areas of pharmaceutical manufacturing. Experts estimate that by 2026 orphan drugs will account for 20% of all prescription drug sales and 30% of the global drug pipeline’s value [393]. The enormous potential does not translate into tangible health benefits due to gaps in access to therapies. This problem is raised in the European pharmaceutical strategy, which emphasizes the need to analyze the reasons for the deferred marketing of centrally authorized products [394]. August 2022 marked the end of the pilot program “Market launch of Centrally Authorized Medicinal Products,” initiated by the EC in cooperation with the EMA, EU countries and potential marketing authorization holders to analyze the problem [395]. In the Visegrad countries, the highest aggregate level of restrictions on access to modern treatment was found for rare diseases. To illustrate, Poland’s average time from registration to reimbursement for all drugs included in the study was 940 days, 3.4 years. The time between registration for an indication and reimbursement decision was longest for new therapies for mucoviscidosis (7.4 years) and diabetes (5.2 years) and shortest for NSCLC (2.3 years) and SMA (1.6 years) [392]. An example outside the list of diseases shown in the report can also be an ultra-rare metabolic disease—ceroid lipofuscinosis neuronal type 2, the treatment of which is financed in selected European countries but not in Poland, even under the procedure of emergency access to drug technology [396]. This situation results in the emigration of the vast majority of newly diagnosed patients to neighbouring Germany [387]. 

The challenges are many, but awareness is definitely growing, and so is the stature of rare diseases. The last decade has brought several ideas, solutions and recommendations for positive change for patients. Many are under the auspices of EURORDIS (European Organization for Rare Diseases), which aims to increase the number of new therapies for rare diseases annually by 3 to 5 times by 2025 while lowering their cost by 3 to 5 times [397]. The UN, the WHO, and IRDiRC are moving in the same direction—rare diseases are an essential link in the chain of sustainable development [5,388,390,391]. A recent Rare 2030 trends study shows that increasing strains on healthcare budgets will force the emergence of new models of care delivery while inequalities in access to treatment and care continue to grow across Europe among people with rare diseases [389]. It is essential to eliminate gaps in access to orphan therapies and increase their supply to develop a consensus that benefits patients, manufacturers and payers. Restricting markets to a narrow customer base limits drug pricing and risk-sharing mechanisms. Under current circumstances, with deadly diseases and often the only treatment options, health emigration seems to have a forced character. This situation prompts intensified discussion of creating supra-state governance rules for rare diseases, including therapy financing. The issue is complex and challenging but has its grounding in the context of rare diseases and will be taken up by the article’s authors in future research. 

## 5. Conclusions

The goal of the study, which was to systematize knowledge on health emigration in rare diseases, mainly using machine learning, was achieved. The main conclusions (the context is described further) are as follows:

- ML allowed us to objectively diagnose a research gap in the area of rare disease management—the issue of health emigration is not directly studied;
- Analysis of grey literature (including industry reports) confirmed the gap;
- The need to define the term “health emigration”;
- Health emigration as a derivative of health inequalities should be included on the social cost side of the rare disease management model at the national and supranational levels;
- The topic of health emigration in rare diseases needs further research at several levels.

The analysis of scientific literature, as well as industry literature, provided answers to the research questions posed. Namely, the application of machine learning in terms of article titles and keywords resulted in extracting the most critical words, among which the following dominated: disease, rare, orphan, drug, patient, access, and health. An in-depth analysis of the latent words made it possible to see the researchers’ concentration around four complementary thematic clusters—the perspectives of the patient, the decision-maker, the manufacturer, and the perspective integrating all stakeholders. The authors found the indicated theme of geographic differentiation inspiring, although requiring further in-depth analysis taking into account the specifics of the areas in question to draw firm conclusions. The review identified researchers’ research interests in rare diseases within the framework of socioeconomic issues but lacked direct threads related to health emigration. Available industry reports point to the problem of ever-increasing inequalities in access to treatment and care in European countries but does not directly address the derivative of these inequalities, i.e., health emigration and the effects it generates. 

Given the diagnosed research gap in the definition, characteristics, and effects of “health emigration”, the authors offer their definition. Health emigration is a trip to another country to live permanently or for a temporary stay caused by the desire to obtain more effective health care not available in the country of origin. This situation is not health tourism, but a permanent/long-term trip since rare diseases tend to be chronic and require long-term treatment. Significantly, the problem affects the whole family, as rare diseases mainly manifest during childhood. Although the decision to emigrate is at the family’s discretion, it often bears the hallmarks of a forced one since remaining in the country would mean death for the patient in the short or long term. It mainly affected families who emigrate, for whom the first therapeutic option, possibly another far more effective one, has appeared on the market. The social and economic costs that emigration entails should be considered in the analysis process for reimbursement decisions. 

Despite increasing awareness in public about rare diseases and their growing prominence in health policies, disparities between countries in implementing pro-patient solutions and funding for treating these diseases are very pronounced. With simultaneous access to information, awareness and knowledge of the patient community, decisions to leave the country to obtain treatment are made frequently. Assuming that the number of orphan therapies implemented will continue to grow without innovative solutions for equal access to reimbursed treatment, the phenomenon of health emigration in rare diseases will increase. 

This study has several limitations in terms of the following:

- The selection of the base of the analyzed literature;
- Tthe supplementation of the analyzes with selected industry reports;
- The lack of references to domestic migration;

The article helped identify a gap in researcher activity on the topic of health emigration in rare diseases; therefore, the article in question only provides suggestions for further research to improve health policy for rare diseases in the future. 

Only articles indexed in databases were used in the analysis: Scopus, the Web of Science and PubMed, which may have caused the authors to omit valuable literature items. To complete the picture, they used an analysis of selected industry reports where, to the best of the authors’ knowledge, issues of health inequality and the gap in access to therapy were addressed. At the same time, these reports are geographically piecemeal, dealing with selected markets and do not analyze the problem of health emigration. A related issue, nevertheless not addressed in this work, is migration and travel within a country. Access to medical services, especially specialized ones, is limited to one/some specialized centres in a country, which reduces the comfort of the chronically treated patient. Managing access to treatment within a country to increase patient comfort is an area for consideration in addressing health needs in rare diseases. Therefore, the authors of the article propose and, in their further research, will address these issues in terms of the scale and effects of emigration on several levels: for the family, the country of origin, the destination country, and the rare disease management system in general. In the future, it is essential to add other languages and sources to the database, such as medical organizations, regional and global health reports, and other publications that are not focused on business and science. 

## Figures and Tables

**Table 1:** Topic modeling using latent semantic indexing (LSI).

Topic	Topic Keywords
1	drug, disease, orphan, rare, patient, treatment, cost, policy, access, medicine
2	drug, disease, rare, orphan, HTA, price, patient, cost, R&D, care
3	drug, HTA, orphan, China, OD, R&D, nice, technology, development, OMP
4	China, HTA, policy, product, designation, rare, insurance, country, FDA, approval
5	medicine, cost, HTA, OD, drug, R&D, orphananesthesia, China, recommendation, nice

Note: Topic evaluation: topic coherence = 0.3841. Black characters mean that the word represents the subject strongly, while red characters mean that the word is not clearly representative of the subject. Source: own elaboration based on 343 references [44,45,46,47,48,49,50,51,52,53,54,55,56,57,58,59,60,61,62,63,64,65,66,67,68,69,70,71,72,73,74,75,76,77,78,79,80,81,82,83,84,85,86,87,88,89,90,91,92,93,94,95,96,97,98,99,100,101,102,103,104,105,106,107,108,109,110,111,112,113,114,115,116,117,118,119,120,121,122,123,124,125,126,127,128,129,130,131,132,133,134,135,136,137,138,139,140,141,142,143,144,145,146,147,148,149,150,151,152,153,154,155,156,157,158,159,160,161,162,163,164,165,166,167,168,169,170,171,172,173,174,175,176,177,178,179,180,181,182,183,184,185,186,187,188,189,190,191,192,193,194,195,196,197,198,199,200,201,202,203,204,205,206,207,208,209,210,211,212,213,214,215,216,217,218,219,220,221,222,223,224,225,226,227,228,229,230,231,232,233,234,235,236,237,238,239,240,241,242,243,244,245,246,247,248,249,250,251,252,253,254,255,256,257,258,259,260,261,262,263,264,265,266,267,268,269,270,271,272,273,274,275,276,277,278,279,280,281,282,283,284,285,286,287,288,289,290,291,292,293,294,295,296,297,298,299,300,301,302,303,304,305,306,307,308,309,310,311,312,313,314,315,316,317,318,319,320,321,322,323,324,325,326,327,328,329,330,331,332,333,334,335,336,337,338,339,340,341,342,343,344,345,346,347,348,349,350,351,352,353,354,355,356,357,358,359,360,361,362,363,364,365,366,367,368,369,370,371,372,373,374,375,376,377,378,379,380,381,382,383,384,385,386].

**Table 2:** Topic modeling using latent semantic indexing (LSI) for countries.

Topic Keywords Map	Topic	Topic Keywords
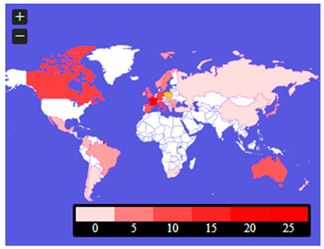	#	case: Poland
	1	drug, country, disease, orphan, requirement, rare, access, GDP, MCDA, HTA
	2	GDP, per, MCDA, cost, OD, capita, annual, Russia, criterion, world
	3	MCDA, GDP, criterion, world, real, Russia, per, Netherlands, framework, search
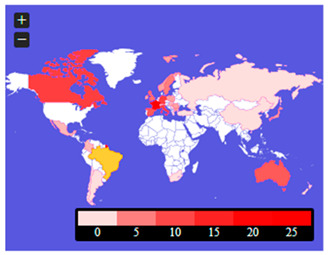	#	case: Brazil
	1	disease, rare, policy, country, patient, legislation, Latin, orphan, clinical
	2	plan, need, law, adoption, analysis, six, strengthen, patient, policy, implementation
	3	drug, policy, regard, LA, market, Latin, orphan, patient, EU, company

Note: topic evaluation: topic coherence (Poland) = 0.4427; topic coherence (Brazil) = 0.4866. Black characters mean that the word represents the subject strongly, while red characters mean that the word is not clearly representative of the subject. Yellow color means selection of the country. Source: own elaboration based on 343 references [44,45,46,47,48,49,50,51,52,53,54,55,56,57,58,59,60,61,62,63,64,65,66,67,68,69,70,71,72,73,74,75,76,77,78,79,80,81,82,83,84,85,86,87,88,89,90,91,92,93,94,95,96,97,98,99,100,101,102,103,104,105,106,107,108,109,110,111,112,113,114,115,116,117,118,119,120,121,122,123,124,125,126,127,128,129,130,131,132,133,134,135,136,137,138,139,140,141,142,143,144,145,146,147,148,149,150,151,152,153,154,155,156,157,158,159,160,161,162,163,164,165,166,167,168,169,170,171,172,173,174,175,176,177,178,179,180,181,182,183,184,185,186,187,188,189,190,191,192,193,194,195,196,197,198,199,200,201,202,203,204,205,206,207,208,209,210,211,212,213,214,215,216,217,218,219,220,221,222,223,224,225,226,227,228,229,230,231,232,233,234,235,236,237,238,239,240,241,242,243,244,245,246,247,248,249,250,251,252,253,254,255,256,257,258,259,260,261,262,263,264,265,266,267,268,269,270,271,272,273,274,275,276,277,278,279,280,281,282,283,284,285,286,287,288,289,290,291,292,293,294,295,296,297,298,299,300,301,302,303,304,305,306,307,308,309,310,311,312,313,314,315,316,317,318,319,320,321,322,323,324,325,326,327,328,329,330,331,332,333,334,335,336,337,338,339,340,341,342,343,344,345,346,347,348,349,350,351,352,353,354,355,356,357,358,359,360,361,362,363,364,365,366,367,368,369,370,371,372,373,374,375,376,377,378,379,380,381,382,383,384,385,386].

**Table 3:** Emotion recognition in publications with a selection of country.

Emotion Profile	Structure of Emotions
All selected countries from Figure 5a	
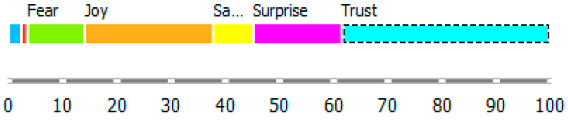	In descending order: turquoise bar means trust (38.52%); orange bar—joy (23.74%); pink—surprise (16.34%); green—fear (10.51%); yellow—sadness (7.39%); blue—anger (2.33%); red—disgust (1.17%)
Poland	
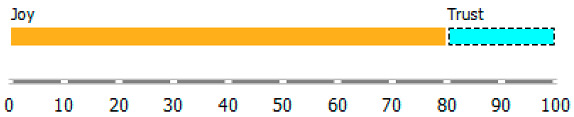	In descending order: orange bar means joy (80%); turquoise—trust (20%)
Brazil	
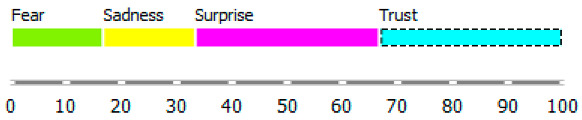	In descending order: turquoise bar means trust (33.33%); pink—surprise (33.33%); yellow—sadness (16.67%); green—fear (16.67%)

Source: own elaboration based on 343 references [44,45,46,47,48,49,50,51,52,53,54,55,56,57,58,59,60,61,62,63,64,65,66,67,68,69,70,71,72,73,74,75,76,77,78,79,80,81,82,83,84,85,86,87,88,89,90,91,92,93,94,95,96,97,98,99,100,101,102,103,104,105,106,107,108,109,110,111,112,113,114,115,116,117,118,119,120,121,122,123,124,125,126,127,128,129,130,131,132,133,134,135,136,137,138,139,140,141,142,143,144,145,146,147,148,149,150,151,152,153,154,155,156,157,158,159,160,161,162,163,164,165,166,167,168,169,170,171,172,173,174,175,176,177,178,179,180,181,182,183,184,185,186,187,188,189,190,191,192,193,194,195,196,197,198,199,200,201,202,203,204,205,206,207,208,209,210,211,212,213,214,215,216,217,218,219,220,221,222,223,224,225,226,227,228,229,230,231,232,233,234,235,236,237,238,239,240,241,242,243,244,245,246,247,248,249,250,251,252,253,254,255,256,257,258,259,260,261,262,263,264,265,266,267,268,269,270,271,272,273,274,275,276,277,278,279,280,281,282,283,284,285,286,287,288,289,290,291,292,293,294,295,296,297,298,299,300,301,302,303,304,305,306,307,308,309,310,311,312,313,314,315,316,317,318,319,320,321,322,323,324,325,326,327,328,329,330,331,332,333,334,335,336,337,338,339,340,341,342,343,344,345,346,347,348,349,350,351,352,353,354,355,356,357,358,359,360,361,362,363,364,365,366,367,368,369,370,371,372,373,374,375,376,377,378,379,380,381,382,383,384,385,386].

## Data Availability

Data are contained within the article.
